# Unraveling Interspecies Differences in the Phase I Hepatic Metabolism of Alternariol and Alternariol Monomethyl Ether: Closing Data Gaps for a Comprehensive Risk Assessment

**DOI:** 10.1021/acs.chemrestox.4c00095

**Published:** 2024-07-19

**Authors:** Eszter Borsos, Elisabeth Varga, Georg Aichinger, Doris Marko

**Affiliations:** † Department of Food Chemistry and Toxicology, Faculty of Chemistry, University of Vienna, Vienna 1090, Austria; ‡ Doctoral School in Chemistry, Faculty of Chemistry, University of Vienna, Vienna 1090, Austria; § Unit Food Hygiene and Technology, Centre for Food Science and Veterinary Public Health, Clinical Department for Farm Animals and Food System Science, University of Veterinary Medicine, Vienna, Vienna 1210, Austria; ∥ Department of Health Sciences and Technology, ETH Zürich, Zürich 8092, Switzerland

## Abstract

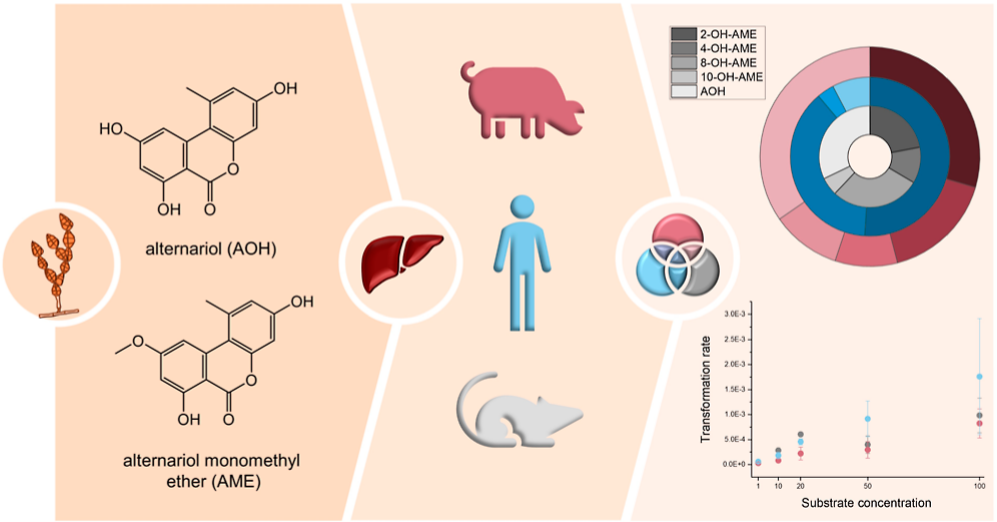

The *Alternaria* mycotoxins alternariol (AOH) and alternariol 9-*O*-monomethyl ether (AME) are pervasive food contaminants known to exert adverse effects in vitro, yet their toxicokinetics remain inadequately understood. Thus, this study endeavors to elucidate the qualitative and quantitative aspects of the phase I metabolism of AOH and AME. To pursue this goal, reduced nicotinamide adenine dinucleotide phosphate (NADPH)-fortified porcine, rat, and human liver microsomes were incubated for 0–10 min with AOH or AME within a concentration range of 1–100 and 1–50 μM, respectively. The decline in the parent toxin concentration was monitored via liquid chromatography coupled to tandem mass spectrometry, whereas coupling to high-resolution mass spectrometry provided insights into the composition of the arising metabolic mixture. The collected quantitative data allowed us to calculate the hepatic intrinsic clearance rates of AOH and AME, marking a notable contribution to the field. Moreover, we unveiled interspecies differences in the pattern and rate of the phase I metabolism of the investigated mycotoxins. The presented findings lay the groundwork for physiologically based toxicokinetic modeling aimed at estimating local concentrations of these mycotoxins in specific organs, enhancing our understanding of their mode of action and adverse health effects.

## Introduction

**Figure 1:**
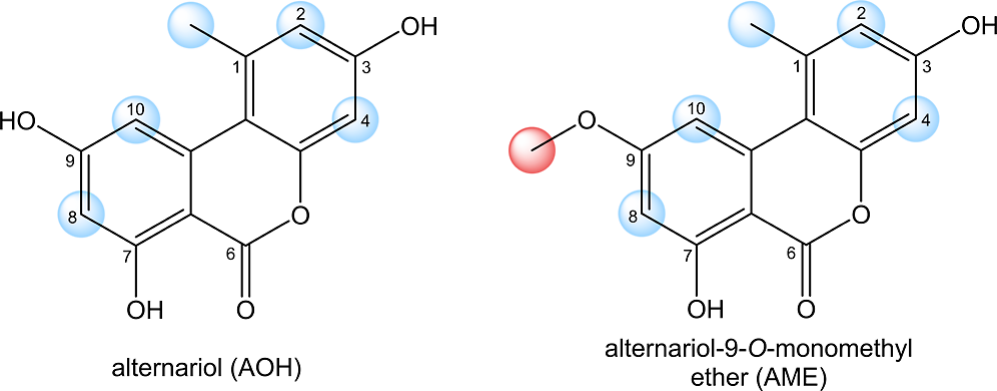
Chemical structures of AOH and AME. Possible hydroxylation sites are marked blue, and the demethylation position is highlighted in red.

Mycotoxins share the ability to exert adverse effects on vertebrates even at low concentrations, despite their diversity in structure and fungus of origin.^1^ Therefore, their entry into the food and feed chain raises significant concerns regarding potential health risks to humans and animals.^2^*Alternaria* fungi form more than 70 secondary metabolites, some of which act as mycotoxins, such as the dibenzo-α-pyrones alternariol (AOH) and alternariol 9-*O*-monomethyl ether (AME; Figure 1). They have been reported to possess cytotoxic,^3−5^ genotoxic and mutagenic,^6−9^ immunosuppressive,^10,11^ and potential endocrine disruptive^12,13^ properties. This complex pattern of often overlaying effects is comprehensively summarized in a recent review by Louro et al.^14^

*Alternaria* species have a wide host range, thriving on grains, fruits, tomatoes, sunflower seeds, olives, etc. Their ubiquity, resilience, and ability to withstand even low temperatures allow them to contaminate foods after harvest and persist during storage or transport, even under refrigeration.^15^ As a result, mycotoxins produced by *Alternaria* molds are frequently detected in various food commodities, often at significant levels.^16^ In response to this, indicative values for AOH and AME in food were proposed in the Commission Recommendation (EU) no. 2022/553,^17^ emphasizing the importance of continuous monitoring of these substances.

Moreover, based on the occurrence and toxicity data of AOH and AME, the European Food Safety Authority stated that the average chronic dietary exposure to these compounds at the upper bound and 95th percentile exceeds the threshold of toxicological concern.^18^ Despite this recognition, there are currently no regulations in place for AOH and AME in food or feed. This underlines the immediate requirement for additional data to enable a comprehensive risk assessment of these emerging mycotoxins.

The most prominent data gap concerns the toxicokinetic behavior of AOH and AME, comprising information on their absorption, distribution, metabolism, and excretion (ADME).^19^ Existing studies suggest a low oral bioavailability for these toxins. For instance, oral exposure to radiolabeled AOH in male NMRI mice showed less than 10% bioavailability, with most of the toxin excreted unchanged in feces.^20^ Similarly, AME was absorbed in less than 10% following oral administration in rats, with the majority excreted in feces.^21^ In vitro studies in Caco-2 cell monolayers support these findings, indicating limited intestinal absorption of AOH and AME, with significant portions undergoing conjugation.^22^ Furthermore, a human biomonitoring study by Krausová et al. detected low levels of AME in the feces of Nigerian infants, suggesting chronic low-level exposure and limited absorption.^23^ Despite these valuable studies, the absorption and intestinal metabolism of these mycotoxins are not yet fully understood, and this uncertainty impairs our ability to predict their concentrations in the liver and other tissues.

When it comes to their biotransformation, existing literature has identified the major metabolites and estimated the extent of biotransformation of AOH and AME in various in vitro and in vivo models.^22,24−29^ Nevertheless, these investigations are limited to one incubation time and one toxin concentration, impeding the calculation of essential kinetic parameters. The absence of such data hinders the development of physiologically based toxicokinetic (PBTK) models for in vitro, in vivo, and cross-species extrapolations. However, these models and extrapolations are essential for quantitative insights into the toxic effects of AOH and AME within the human body by enabling the assessment of adverse health effects over time, identification of target organs, and determination of potentially susceptible populations to these fungal metabolites.^30,31^

As a consequential step toward the overall aim detailed above, this work addresses the hepatic metabolism kinetics of AOH and AME using liver microsomes (LMs) across various mammalian species. The main objective was to capture interspecies variations and determine kinetic parameters through incubation studies conducted in porcine, human, and rat LMs fortified with reduced nicotinamide adenine dinucleotide phosphate (NADPH). The acquired hepatic intrinsic clearance values will hopefully contribute to an overarching risk assessment of these ubiquitous xenobiotics.

## Experimental Procedures

### Biological Materials

Pooled human LMs and pooled Sprague–Dawley rat hepatic microsomes, both originating from male donors, were purchased from Sigma-Aldrich (St. Louis, MO, USA) and Gibco, Thermo Fisher Scientific (Waltham, MA, USA), respectively. Porcine LMs were prepared according to an in-house-developed protocol based on the publication of Knights et al.^32^ The liver of an approximately four-month-old female “Large White” (or “German Edelschwein”) pig was obtained from a local slaughterhouse. After slaughtering, the liver was placed on ice for about an hour, kept in ice-cold 50 mM tris(hydroxymethyl)aminomethane hydrochloride (Tris–HCl) (pH 7.4) for an additional hour while being transported, and processed immediately.

### Chemicals

Dimethyl sulfoxide (DMSO) and Tris–HCl were purchased from Carl Roth GmbH & Co. (Karlsruhe, Germany). AOH and AME from *Alternaria* species, as well as liquid chromatography coupled with mass spectrometry (LC–MS)-grade ammonium acetate and ammonium hydroxide solutions, were purchased from Sigma-Aldrich (St. Louis, MO, USA). The single certified analytical standards of AOH and AME were produced by Romer Labs Diagnostic GmbH (Tulln, Austria), and the *Alternaria* reference mixture^28,33^ was provided by Dr. Hannes Puntscher. The tetrasodium salt of NADPH and l-ascorbic acid were purchased from Merck (Darmstadt, Germany). CHROMASOLV LC–MS-grade acetonitrile and methanol (Honeywell Riedel-de Haën, Seelze, Germany) served as an eluent or extraction solvent for analysis by LC–MS. Finally, HiPerSolv CHROMANORM LC–MS-grade water was purchased from VWR International (Radnor, PA, USA).

### Microsomal Incubations

The kinetics of the phase I metabolism of AOH and AME was investigated by incubating human, rat (both 1 mg protein/mL), and porcine LMs (2 mg protein/mL) with 1, 10, 20, 50, and 100 μM AOH or 1, 10, 20, and 50 μM AME based on the protocol by Al-Subeihi et al.^34^ The incubation solution contained a final concentration of 3 mM NADPH and 1 mM ascorbic acid in 200 mM Tris–HCl (pH 7.4). After preincubation (37 °C, 1 min, mixing at 250–300 rpm), the toxin stock was added in a 1:100 dilution, resulting in a total DMSO content of 1% and the desired toxin concentration. DMSO was selected for this assay because it is the least inhibitory of the solvents studied by Busby et al. on CYP1A1,^35^ the primary enzyme involved in the hydroxylation of AOH and AME.^26^ The well-established solubility of the test compounds in DMSO and DMSO-containing aqueous buffers facilitated laboratory work and ensured consistency with existing literature, thereby improving the comparability of results across studies, including those of Pfeiffer and colleagues.^25^ In a preliminary investigation, incubation times of 5 and 10 min were chosen because they fall within the linear range of the transformation rate–time relationship, thereby ensuring initial rate conditions (Figure S1). When the selected incubation time (0–10 min) passed, one part of the sample was pipetted into two parts of ice-cold extraction solvent (acetonitrile–methanol, 1:1, v/v) for reaction termination. Subsequently, the samples were placed into the freezer (−20 °C) for at least an hour, centrifuged (15 min, 18,000*g*, 4 °C), and further diluted in methanol–water (3:7, v/v) prior to LC–MS measurements. In the solvent control, the toxin was substituted with DMSO in the incubation solution. Control incubations were performed without the cofactor NADPH or with heat-inactivated microsomes (98 °C, 10 min, mixing at 300 rpm).

### Analysis with High-Performance Liquid Chromatography Coupled to Tandem Mass Spectrometry

The quantification of selected analytes using external calibration (MS parameters listed in Supporting Information Table S1) was conducted on a high-performance liquid chromatographic system (Dionex UltiMate 3000 UHPLC, Dionex Softron GmbH, Germering, Germany) coupled to a triple quadrupole mass spectrometer (TSQ Vantage, Thermo Fisher Scientific, Waltham, MA, USA), equipped with a heated electrospray ionization (ESI) interface. The applied analytical method is based on Puntscher et al.^28,36^ with slight modifications, which are specified below.

In brief, the Ascentis Express C18 column (10 cm × 2.1 mm, 2.7 μm, Supelco, Munich, DE), equipped with the Phenomenex SecurityGuard (C18 Cartridges, 4 × 2.0 mm ID, Phenomenex Ltd. Deutschland, Aschaffenburg, Germany), served as the stationary phase. Eluent A was aqueous ammonium acetate (5 mM, pH adjusted to 8.6 with a 25% ammonia solution), and eluent B was methanol. During the first minute, the column was kept at 30% eluent B. Subsequently, the eluent B content was linearly raised to 100% within 6 min. Thereafter, the column was washed with 100% eluent B for 1.5 min. Lastly, the column was re-equilibrated at the initial conditions, reaching an overall run time of 10 min. The autosampler compartment was kept at 10 °C, while the column oven temperature was maintained at 30 °C. The tandem mass spectrometer was operated in multiple reaction monitoring mode using negative ionization, detecting the compounds of interest in their deprotonated forms. Data acquisition and evaluation were performed using the Thermo XCalibur (v 4.0.27.42) and TraceFinder (v 3.3.358.0) software, both from the company Thermo Fisher Scientific (Waltham, MA, USA).

### Statistics and Determination of the Kinetic Constants

The obtained concentration values were tested for normality, and one-way analysis of variance (ANOVA), followed by Fisher’s least significant difference (LSD) posthoc test, was used to test significant differences. Curve fitting to Michaelis–Menten kinetics and the calculation of kinetic parameters were performed using the Levenberg–Marquardt algorithm in OriginPro 2021b (v. 9.8.5.212).

### HR-MS Measurements

Pooled high-resolution MS (HR-MS) samples were analyzed on a Vanquish UHPLC system (Thermo Fisher Scientific, Waltham, MA, USA) connected to a dual-pressure linear trap-quadrupole Orbitrap mass analyzer (Velos ETD, Thermo Fisher Scientific, Waltham, MA, USA). The heated ESI interface (source heater temperature: 400 °C) was operated in both positive and negative modes. The mobile and stationary phases were the same as those for the targeted analysis. The column compartment temperature was kept at 40 °C. A multistep gradient was applied at a flow rate of 0.4 mL/min and started with rinsing the column for 1 min at 10% eluent B. Then, the organic content was linearly raised to 100% until the 11th minute. Subsequently, the column was purged with 100% eluent B for two additional minutes. Finally, the initial eluent composition was reset between minutes 13 and 13.5, followed by a 2 min re-equilibration under these conditions, resulting in a total run time of 15.5 min. Data acquisition and evaluation were performed with the software Skyline (v. 21.1.0.146, MacCoss Lab, Department of Genome Sciences, University of Washington, Seattle, WA, USA), XCalibur (v. 2.2SP1.48), and Chromeleon (v. 7.2.6.; both Thermo Fisher Scientific, Waltham, MA, USA).

As no analytical standards are currently available for the hydroxy (OH) metabolites of AOH and AME, an *Alternaria* reference mixture containing 4-OH-AOH and 4-OH-AME was used to enable associating peaks with their respective phase I metabolites. Further peak assignments relied on the elution order obtained under comparable reversed-phase liquid chromatographic conditions by Pfeiffer and co-workers^25^ while acknowledging the possibility of minor variations in this order.

## Results

### Parent Toxin Loss

**Figure 2:**
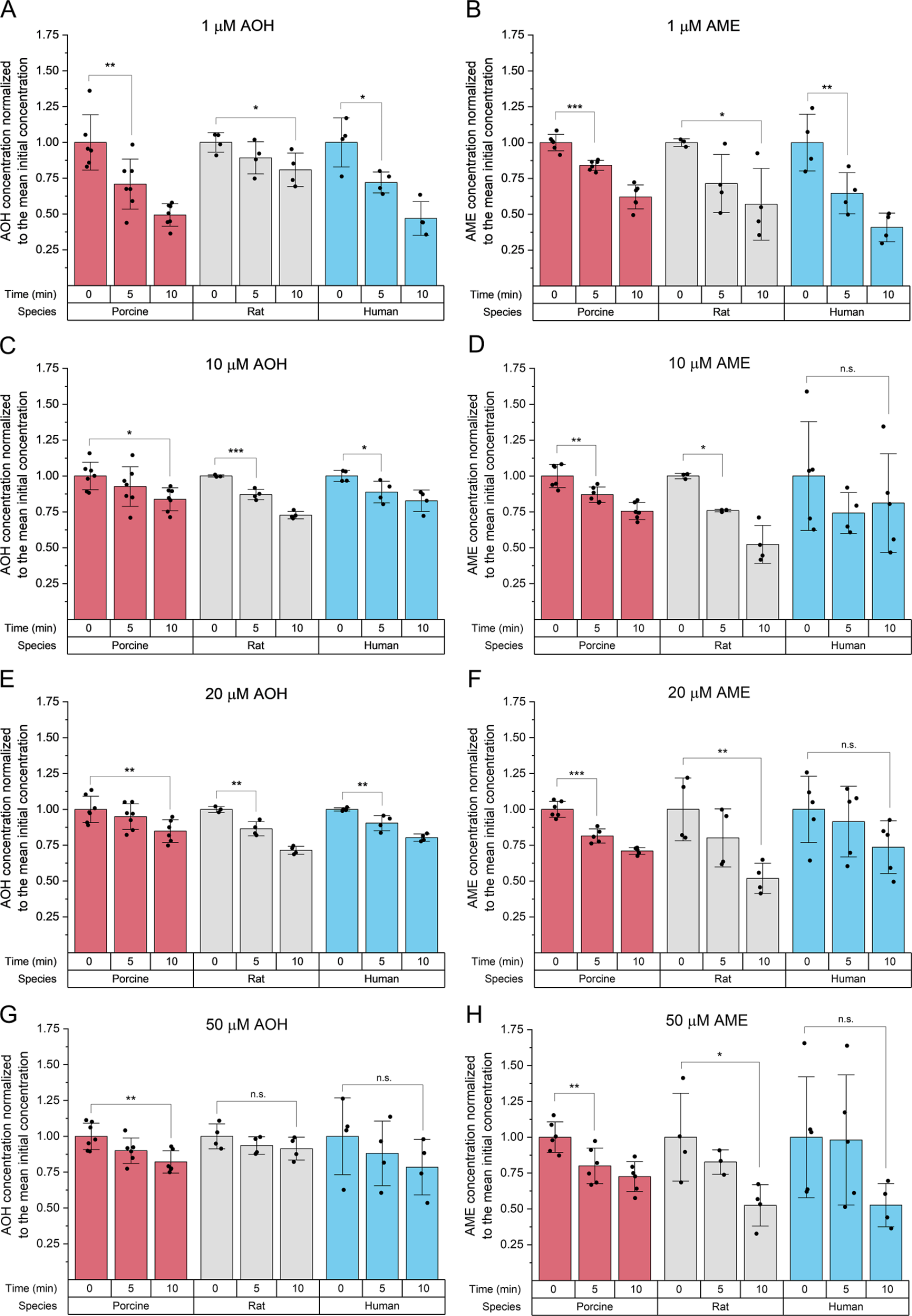
Interspecies differences in the toxin level decrease after the incubation of LMs with 1–50 μM AOH and AME. Columns represent the means ± SD of at least three independent experiments. After testing for normality, one-way ANOVA, followed by Fisher’s LSD posthoc test, was used to detect significant differences. The significance levels are marked as follows: n.s. → no significant difference; * → 0.01 < *p* < 0.05; ** → 0.001 < *p* < 0.01; and *** → *p* < 0.001.

In all species tested, the initial concentrations of AOH and AME were similar (Supporting Information Table S2) and showed a declining trend after 10 min of incubation, which is statistically significant for most of the conditions studied (Figure 2 and Supporting Information Figure S2). However, treating human LMs with AME in concentrations higher than 1 μM did not statistically reduce the initial toxin level (Figure 2D,F,H) due to the higher standard deviation (SD) of the obtained results. Furthermore, the toxin concentration remained constant in the NADPH-free and heat-treated controls (Figure S3). This observation confirms that the reduction in toxin levels depicted in Figure 2 is attributed to a metabolic reaction between the hepatic microsomal enzymes and AOH or AME, with NADPH acting as an essential cofactor, excluding other physicochemical phenomena contributing to this depletion.

### Metabolite Identification

**Figure 3:**
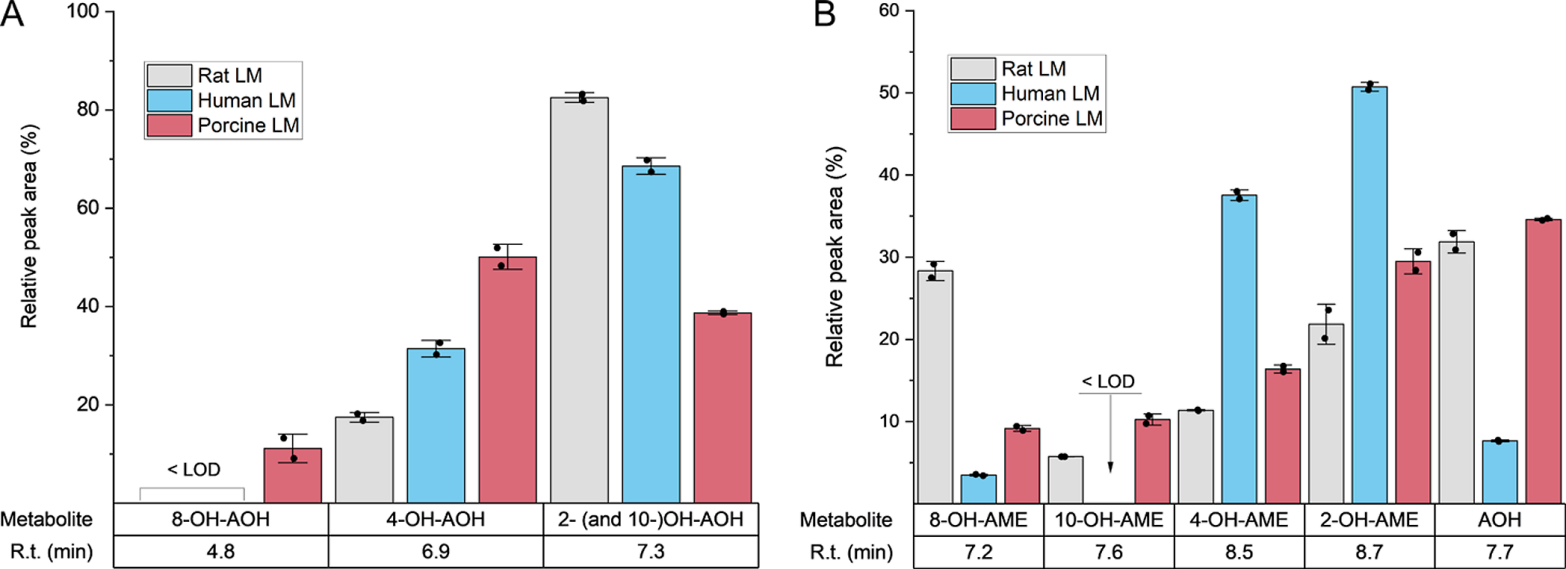
Interspecies differences in the pattern of oxidative metabolites occurred in LMs after a 10 min incubation with 10 μM AOH (A) or AME (B). The depicted values are normalized to the sum of the measured peak areas per species. Each bar shows the average of two measurements of one pooled sample ± SD. The abbreviation < LOD stands for analytes below the limit of detection, which applies for 8-OH-AOH in rat and human LMs as well as 10-OH-AME in human LMs. R.t. stands for retention time.

The HR-MS measurements of pooled incubation samples enabled us to identify the main phase I metabolites of AOH and AME in the investigated species and unravel potentially occurring interspecies differences in their relative abundance. Based on the elution order of Pfeiffer et al.,^25^ we hypothesized 2- and 10-OH-AOH to coelute in our method. Moreover, we assumed a similar ionization efficacy for all of the analytes by comparing their abundance in the metabolic mixture based on their relative peak area. Considering these presumptions, the sum of 2- and 10-OH-AOH seems to be the highest peak in rat and human LMs when incubating with AOH (Figure 3A). In porcine LMs, 4-OH-AOH is the primary oxidative metabolite, followed by the sum of 2- and 10-OH-AOH and small amounts of 8-OH-AOH (see exemplary chromatogram in Supporting Information Figure S8).

In human LMs, 4- and 2-OH-AME are the most abundant phase I metabolites of AME, and only small amounts of 8-OH-AME and AOH occur (Figure 3B). In contrast, AOH is one of the main metabolites in rat and porcine LMs, accompanied by 8-OH-AME in the case of rat LMs and 2-OH-AME in porcine LMs (see the chromatogram in Supporting Information Figure S9). Besides hydroxylated AME, traces of OH-AOH metabolites were detectable in the samples exposed to AME, pointing out that AOH occurring as a hepatic metabolization product of AME rapidly undergoes further functionalization in the presence of microsomal enzymes.

### Determination of the Kinetic Constants

The transformation rate–toxin concentration data points were subjected to linear regression and Michaelis–Menten nonlinear curve fitting (Figures S4 and S5). The obtained kinetic parameters, such as the Michaelis constant (K_m_) and the maximum transformation rate (*V*_max_), are summarized in Table 1.

**Table 1:** Kinetic Parameters Estimated by the Michaelis–Menten Model or Linear Regression for Describing the Metabolism of AOH and AME in Hepatic Microsomes within 10 min^a^

toxin	species	Michaelis–Menten model				linear regression	
		K_m_^b^ (μM)	*V*_max_^c^ (pmol/mg/min)	CL_int_^d^ (mL/mg × min)	R^2^	CL_int_^d^ (mL/mg × min)	R^2^
AOH	pig	*6.72* × *10*^*2*^	*5.73* × *10*^*3*^	*8.52* × *10*^–*3*^	*0.8062*	6.56 × 10^–3^	0.9442
	rat	7.87 × 10^1^	2.83 × 10^3^	3.59 × 10^–2^	0.9872	2.99 × 10^–2^	0.9992
	human	1.31 × 10^2^	3.37 × 10^3^	2.58 × 10^–2^	0.9195	1.99 × 10^–2^	0.9790
AME	pig	3.50 × 10^15^	6.84 × 10^16^	1.95 × 10^–2^	0.9839	1.90 × 10^–2^	0.9905
	rat	1.67 × 10^15^	7.57 × 10^16^	4.52 × 10^–2^	0.9970	4.62 × 10^–2^	0.9985
	human	*2.94* × *10*^*1*^	*7.7*2 × *10*^*2*^	*2.63* × *10*^*–2*^	*0.7902*	1.58 × 10^–2^	0.9980

a Rows marked with italics highlight models with a lower coefficient of determination (R^2^) than 0.9. The statistical significance of the difference between all possible species combinations was assessed using a two-sample *t*-test with a significance level (alpha) set at 0.05. No statistically significant interspecies differences were found.

b K_m_ represents the Michaelis constant.

c *V*_max_ represents the maximum transformation rate.

d CL_int_ describes the intrinsic clearance of the investigated substances.

The ratio between the resulting *V*_max_ and K_m_ values—often referred to as the in vitro intrinsic clearance—provides a relative analysis of the metabolization efficiencies.^37^ With both curve-fitting methods, AOH seems to be metabolized the slowest in pigs, followed by humans and rats. The phase I biotransformation efficacy order for AME is similar to that of AOH according to the Michaelis–Menten model. However, when it comes to the intrinsic clearance values determined via linear regression in the lower concentration range, this order changes slightly, declaring humans the slowest species, followed by pigs and rats.

## Discussion

### Parent Toxin Loss

Incorporating the LMs of various species in our study was crucial to elucidate the interspecies differences in the metabolism of the investigated xenobiotics—a phenomenon long recognized in the field.^38^ While data obtained from human LMs served to assess the risk of AOH and AME to the human population, the LMs of rats were included due to their status as one of the few species with available in vivo data on the ADME of *Alternaria* toxins.^28^ Finally, the substantial exposure of farm livestock to these toxins through feed ingestion^16^ justifies the inclusion of porcine LMs in this work.

The most relevant aspects of comparing the obtained time-dependency data are the remaining toxin level and the time point at which a significant decrease in the initial toxin concentration is reached. Notably, high amounts of parent compounds remained unchanged under our assay conditions in all three species (above approximately 50%). This observation indicates that the phase I biotransformation of AOH and AME could not entirely diminish the adverse effects of the parent compounds, even if the metabolites were innocuous. Moreover, it raises the possibility of combinatory effects between the initial compounds and their metabolites, as previously reported in the case of other mycotoxins, such as zearalenone.^39^

Significant decreases in the case of AOH were observed after 5 min (1 μM for all three species; 10 and 20 μM for rat and human species) or 10 min (10, 20, and 50 μM for porcine LMs), whereas no significant decreases were observed for 50 μM AOH in the case of rat and human species within 10 min (Figures 2 and S6). A similar situation was observed for AME, but overall, AME appears to be metabolized more rapidly in all three species than AOH (Table 1), and lower toxin levels were observed after a 10 min incubation (Figures 2 and S7). However, it is essential to acknowledge that variations between the results obtained in different species may arise from several factors beyond interspecies differences. For instance, while the porcine liver microsomal fractions we used originated from a single female specimen, the pooled human and rat LMs were derived from male donors. Consequently, interindividual and intergender variations cannot be excluded as potential contributors to the observed deviations between the data sets of different species. Moreover, the applied concentration of the microsomal fractions differs—while 1 mg/mL from the rat and human LMs was applied, 2 mg/mL was used in the case of porcine LMs. Nevertheless, the transformation rate values and the intrinsic clearance data calculated thereof overcome this inconsistency in the assay conditions and provide a more reliable and well-established basis for interspecies comparisons.

### Metabolite Identification

The pattern of the occurring phase I metabolites of AOH and AME shows immense differences among the tested mammalian species (Figure 3), probably due to species-specific variations in the expression and activity of the metabolic enzymes involved in the biotransformation of these exogenous compounds.^40^ Despite the limitations of HR-MS quantification due to the lack of analytical standards, these results validly emphasize the importance of considering interspecies differences in xenobiotic metabolism in toxicological evaluations. As demonstrated, beyond the biotransformation rate, the pattern of the occurring metabolites might vary across different mammalian species as well.

Although the concentration (50 vs 10 μM) and incubation time (40 vs 10 min) in this work differed from the experimental setup of Pfeiffer et al.,^25^ the pattern of metabolites is tendentiously comparable in both studies. This observation might suggest that both conditions were in a concentration range where none of the cytochrome P450 (CYP) enzyme isoforms were saturated, causing the relative ratio of the transformation rates per isoform to tend to be constant.

Notably, even within the same species, AOH and AME show substantial deviations in their hydroxylation patterns during oxidative metabolism despite their minimal structural differences. For example, in porcine LMs, 50% of the hydroxylated AOH is conjugated at position 4, less than 20% of 4-OH-AME is produced, and the 2-OH hydroxylation dominates with 30% in the case of AME (Figure 3). This finding demonstrates that even subtle alterations in the chemical structure can profoundly influence the toxicokinetics of a compound, potentially also affecting its overall toxic effect via the formation of distinct metabolites with differing toxicodynamics. This underscores the necessity of testing each substance of question separately during hazard characterization.

It is well-known that phase I xenobiotic metabolism can sometimes lead to the formation of more toxic substances than the parent compound. This issue has already been addressed in the case of 4-OH-AOH and 4-OH-AME in a study on esophageal cells by Tiessen et al.^33^ They found that the 4-hydroxylated derivatives showed less pronounced genotoxic effects than their parent compounds, despite the newly generated catechol structure, probably due to poor cellular uptake resulting from their enhanced polarity. Similarly, 4-hydroxylation was shown to also attenuate the estrogenic properties of AOH and AME.^12^ However, these catechols appear susceptible to subsequent methylation, yielding structures with potential affinity for estrogen receptors, thus restoring or enhancing the estrogenicity of the hydroxylation products.^12,33^ Since each metabolite may exert different adverse health effects, further efforts are necessary to investigate the toxicity of the other oxidative metabolites for a comprehensive risk assessment.

### Determination of the Kinetic Constants

The intrinsic clearance data summarized in Table 1 offer valuable insights into the kinetics of the AOH and AME metabolisms. Although interspecies differences were found to be nonsignificant upon testing, tendencies in the obtained intrinsic clearance values can still be observed. Specifically, considering the phase I metabolism pace of AOH, rat and human LMs exhibited comparable rates, while porcine LMs showed slower metabolism. Conversely, the phase I functionalization of AME appeared to be more similar in humans and pigs, while rats seemed to metabolize this compound slightly faster (Table 1). Overall, these data serve as potential input parameters for a future PBTK model. It is noteworthy to mention that the delivered kinetic data describe solely the phase I metabolism of these mycotoxins, which are also known to undergo phase II conjugation reactions.^27,28^ Therefore, further investigations are warranted to elucidate the kinetics of the phase II metabolism of AOH and AME, providing an exhaustive description of their metabolic kinetics.

The Michaelis–Menten equation represents an enzyme kinetic model that is suitable and widely applied to quantitatively describe the metabolism of xenobiotics in LMs or S9 fractions. However, there are a few limitations to be considered during the experimental design and interpretation of the results. First, the approach assumes steady-state conditions, indicating a constant concentration of the enzyme–substrate complex and only a negligible change in the substrate concentration.^41^ Thus, we attempted to ensure a low transformation rate of the substrate through short incubation times.

In addition, it has been reported that some uridine diphosphate (UDP)-glucuronosyltransferases (UGT) and, more relevantly, CYP enzymes show atypical kinetic profiles.^42^ Thus, beyond the hyperbolic curve based on the Michaelis–Menten equation, a linear curve was fitted to the acquired data points in reasonable concentration ranges. However, linear regression not only aligns with a simplified Michaelis–Menten model but also extends to cover the lower concentration range of enzyme reactions that follow non-Michaelis–Menten kinetics as several of these curves—such as substrate inhibition—exhibit a linear range. Furthermore, the linear regression approach is considered more reliable than the Michaelis–Menten model when the reaction does not reach saturation, as is particularly the case for AME in porcine and rat LMs (Supporting Information Figure S5) or AOH in porcine LMs (Supporting Information Figure S4). In these instances, the kinetic parameters determined by the linear regression method should be used for subsequent PBK modeling. Despite the listed limitations, this approach enabled us to elucidate subtle differences in the biotransformation rates of AOH and AME within one species. More notably, the captured interspecies differences in the pace of the phase I metabolism of these mycotoxins highlight the importance of utilizing species-specific in vitro models for each research question. Otherwise, the adverse effects exerted by exogenous compounds on human or animal health might be underestimated. Furthermore, this approach allowed us to deliver quantitative information about the phase I kinetics of AOH and AME, filling a data gap on the toxicokinetic behavior of these emerging mycotoxins. The gathered data may build a foundation for conducting comprehensive risk assessments.

## Conclusions

In conclusion, our study offers valuable insights into the hepatic metabolism of *Alternaria* toxins AOH and AME across various mammalian species. We unveiled significant interspecies differences in the rate of their biotransformation and the composition of the resulting metabolic mixture.

Exploring these differences underscores the intricate nature of xenobiotic biotransformation processes and highlights the necessity for employing species-specific in vitro models in toxicological studies. Moreover, the delivered kinetic constants provide a robust foundation for forthcoming investigations. Integrating our quantitative data into future PBTK models presents a promising strategy for estimating organ-specific concentrations of AOH and AME and for a more accurate description of their absorption, distribution, metabolism, and excretion in humans.

Overall, the acquired data set fills critical gaps in understanding the toxicokinetic behavior of AOH and AME, laying the groundwork for a more comprehensive risk assessment. Through improved insights into the metabolism of these ubiquitous food contaminants, we take another step toward better safeguarding of human and animal health from their detrimental effects.

## Supporting Information Available

Additional experimental details, presenting MS parameters, results of control incubation experiments, percentage decrease of toxin levels, Michaelis–Menten curve fittings, and exemplary chromatograms (PDF)

## Abbreviations

ADME: absorption, distribution, metabolism, and excretion
AME: alternariol 9-*O*-monomethyl ether
ANOVA: analysis of variance
AOH: alternariol
Cl_int_: intrinsic clearance
CYP: cytochrome P450
DMSO: dimethyl sulfoxide
EFSA: European Food Safety Authority
ESI: electrospray ionization
HR-MS: high-resolution mass spectrometry
K_m_: Michaelis constant
LC–MS: liquid chromatography–mass spectrometry
LM: liver microsome
LOD: limit of detection
LSD: least significant difference
NADPH: reduced nicotinamide adenine dinucleotide phosphate
PBTK: physiologically based toxicokinetic
SD: standard deviation
Tris–HCl: tris(hydroxymethyl)aminomethane hydrochloride
UDP: uridine diphosphate
UGT: UDP-glucuronosyltransferases
*V*_max_: maximum transformation rate
