# Supplementary material for: Unraveling Interspecies Differences in the Phase I Hepatic Metabolism of Alternariol and Alternariol Monomethyl Ether: Closing Data Gaps for a Comprehensive Risk Assessment
Source: Chem Res Toxicol. 2024 Jul 19;37(8):1356–63. doi: 10.1021/acs.chemrestox.4c00095 (PMC11337205; doi:10.1021/acs.chemrestox.4c00095)
Supplement: Supplementary file 1 — tx4c00095_si_001.pdf [file tx4c00095_si_001.pdf]

Supporting Information

Unraveling interspecies differences in the phase I hepatic metabolism of alternariol and alternariol monomethyl ether: closing data gaps for a comprehensive risk assessment

Eszter Borsos[†,‡], *Elisabeth Varga*[†,§,*], *Georg Aichinger*[†,$], *Doris Marko*[†]

[†] Department of Food Chemistry and Toxicology, Faculty of Chemistry, University of Vienna, 1090 Vienna, Austria;

[‡] Doctoral School in Chemistry, Faculty of Chemistry, University of Vienna, 1090 Vienna, Austria;

[§] Unit Food Hygiene and Technology, Centre for Food Science and Veterinary Public Health, Clinical Department for Farm Animals and Food System Science, University of Veterinary Medicine, Vienna, 1210 Vienna, Austria;

[$] Department of Health Sciences and Technology ETH Zürich, 8092 Zürich, Switzerland;

*elisabeth.varga@vetmeduni.ac.at

KEYWORDS: Phase 1 metabolism; *Alternaria*; mycotoxin; kinetics; alternariol; alternariol monomethyl ether

Contents

Table S1: Mass spectrometric parameters of the analytes alternariol (AOH) and alternariol monomethyl ether (AME) quantified via HPLC-MS/MS S3

Figure S1: Determining the linear range in the transformation rate – incubation time relation. S3

Figure S2. Interspecies differences in the toxin level decrease after the incubation of liver microsomes with 100 µM AOH........ S4

Figure S3. Incubation of porcine, rat, and human liver microsomes with 10 µM AOH and AME under different conditions. S5

Table S2: Measured AOH and AME concentrations at the time point 0 in porcine, rat, and human liver microsomes S6

Figure S4. *In vitro* phase I metabolization rates in liver microsomes after 10 minutes of AOH exposure. S7

Figure S5. *In vitro* phase I metabolization rates in liver microsomes after 10 minutes of AME exposure. S8

Figure S6: Percentage decrease of AOH in liver microsomes, with an incubation time of 5-10 minutes........ S9

Figure S7. Percentage decrease of AME in liver microsomes, with an incubation time of 5-10 minutes........ S10

Figure S8: Exemplary chromatograms of monohydroxylated AOH metabolites after a 10-minute incubation of 10 µM AOH with porcine, rat, and human liver microsomes......... S11

Figure S9: Exemplary chromatograms of monohydroxylated AME metabolites after a 10-minute incubation of 10 µM AME with porcine, rat, and human liver microsomes. S12

Table S1: Mass spectrometric parameters of the analytes alternariol (AOH) and alternariol monomethyl ether (AME) quantified via HPLC-MS/MS

Analyte	Precursor ion	S-lens	Product ions				Retention time
			Quantifier		Qualifier		
	(*m/z*)	(V)	(*m/z*)	Collision energy (V)	(*m/z*)	Collision energy (V)	(min)
AOH	257.1	70	215.1	27	147.1	33	4.9
AME	271.1	73	256.1	23	227.1	38	6.4

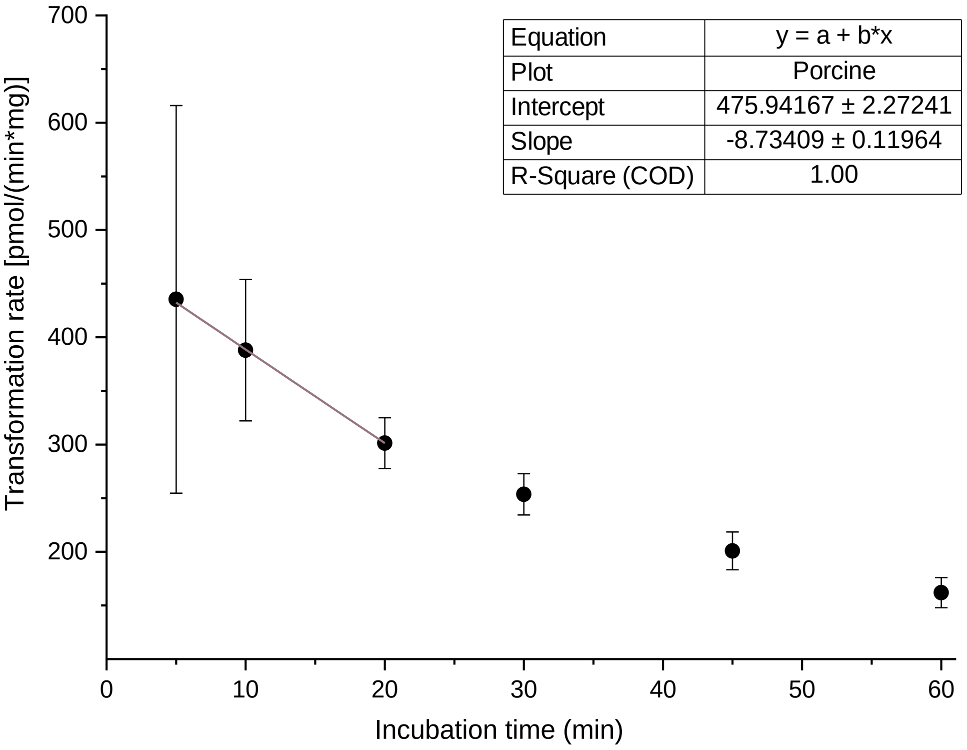

Figure S1: Determining the linear range in the transformation rate – incubation time relation. Example on the data points of incubating porcine liver microsomes with 20 µM AME. Each data point represents mean values of 3-6 independent experiments of one biological replicate ± standard deviation.

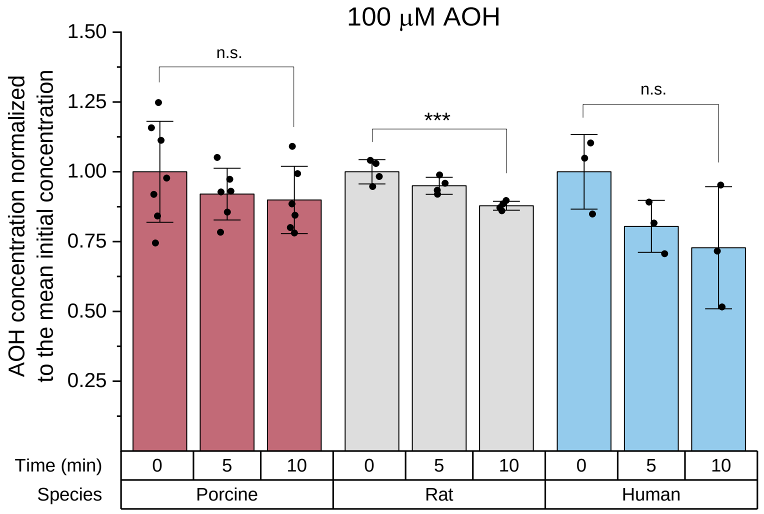

Figure S2. Interspecies differences in the toxin level decrease after the incubation of liver microsomes with 100 μM AOH. Columns represent means ± SD of at least three independent experiments. After testing for normality, one-way ANOVA, followed by Fisher's LSD post-hoc test was used to detect significant differences. The significance levels are marked as follows: n.s. → no significant difference; * → $0.01 < p < 0.05$; ** → $0.001 < p < 0.01$; *** → $p < 0.001$.

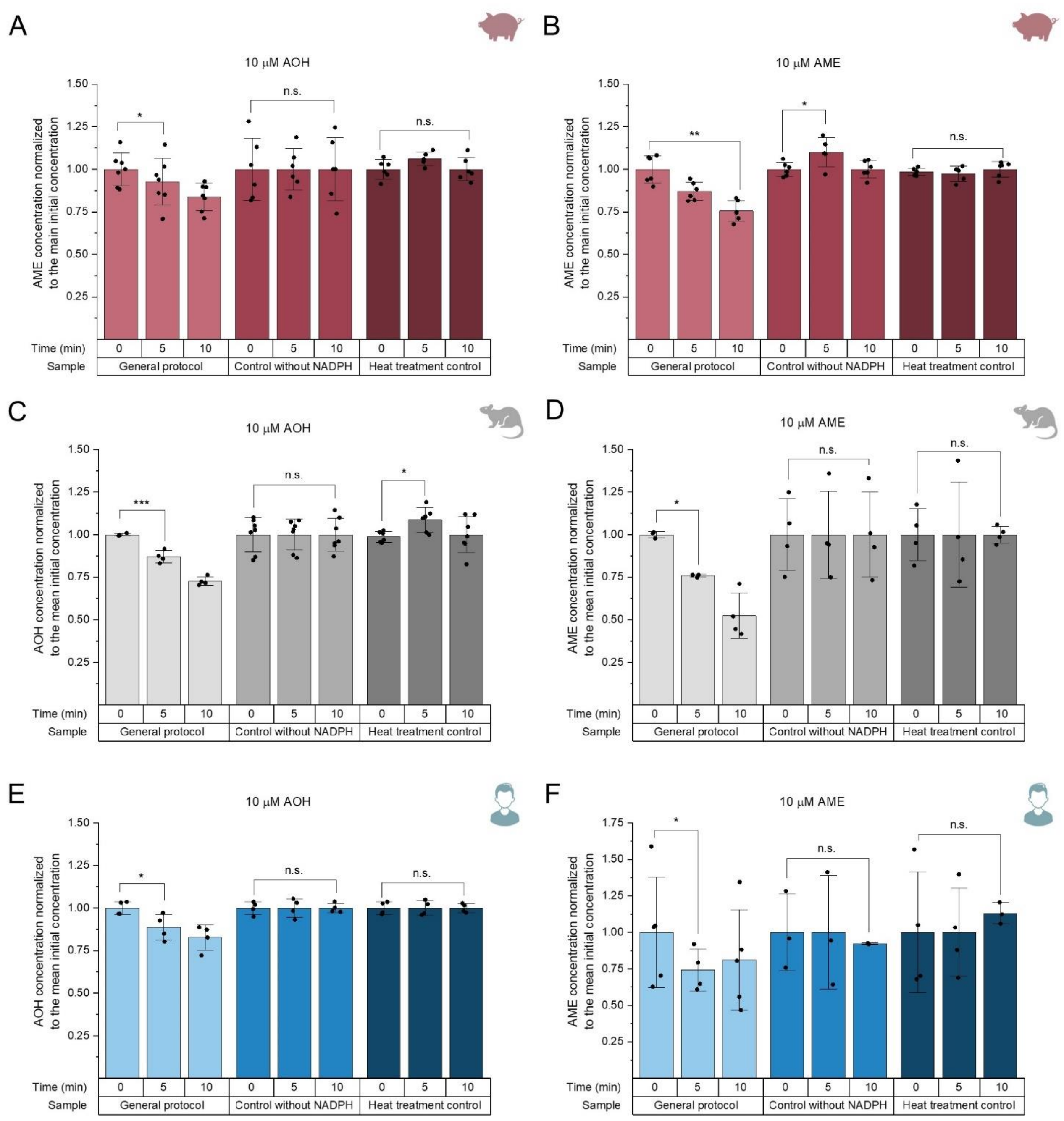

Figure S3. Incubation of porcine, rat, and human liver microsomes with 10 μM AOH and AME under different conditions. Columns represent means ± SD of at least three independent experiments. After testing for normality, one-way ANOVA, followed by Fisher's LSD post-hoc test was used to detect significant differences. The significance levels are marked as follows: n.s. → no significant difference; * → $0.01 < p < 0.05$; ** → $0.001 < p < 0.01$; *** → $p < 0.001$.

Table S2: Measured AOH and AME concentrations at the time point 0 in porcine, rat, and human liver microsomes

Nominal concentration (μM)	Measured initial AOH concentration (μM)			Measured initial AME concentration (μM)		
	Porcine LM	Rat LM	Human LM	Porcine LM	Rat LM	Human LM
1	1.5 ± 0.3	1.1 ± 0.1	1.1 ± 0.2	1.5 ± 0.1	0.7 ± 0.0	0.5 ± 0.1
10	12.1 ± 1.2	9.5 ± 0.1	10.5 ± 0.4	14.5 ± 1.2	8.4 ± 0.2	8.5 ± 3.2
20	21.7 ± 2.0	20.8 ± 0.4	23.2 ± 0.3	27.3 ± 1.5	16.9 ± 3.7	15.8 ± 3.7
50	56.6 ± 5.2	46.6 ±4.0	42.5 ± 11.4	72.6 ± 7.7	61.4 ± 18.8	54.2 ± 22.9
100	114.6 ± 20.7	80.8 ± 3.5	80.7 ± 10.8	not studied due to limitations in aqueous solubility		

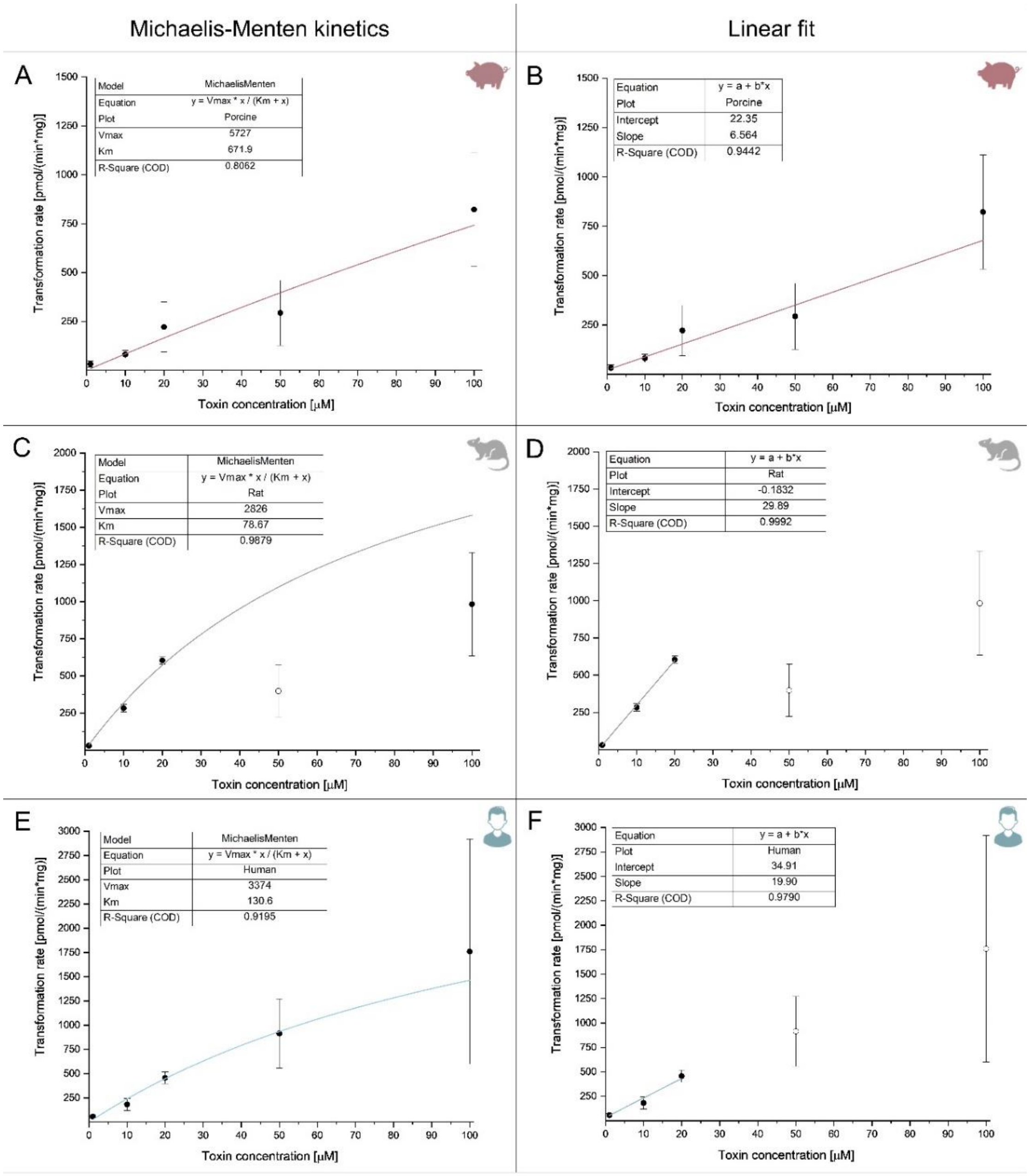

Figure S4. *In vitro* phase I metabolization rates in liver microsomes after 10 minutes of AOH exposure. Dots represent means ± SD of at least three independent experiments, with transformation rates normalized to the microsomal protein content plotted against the initial toxin concentration. Data points represented by hollow circles were excluded from the curve-fitting process.

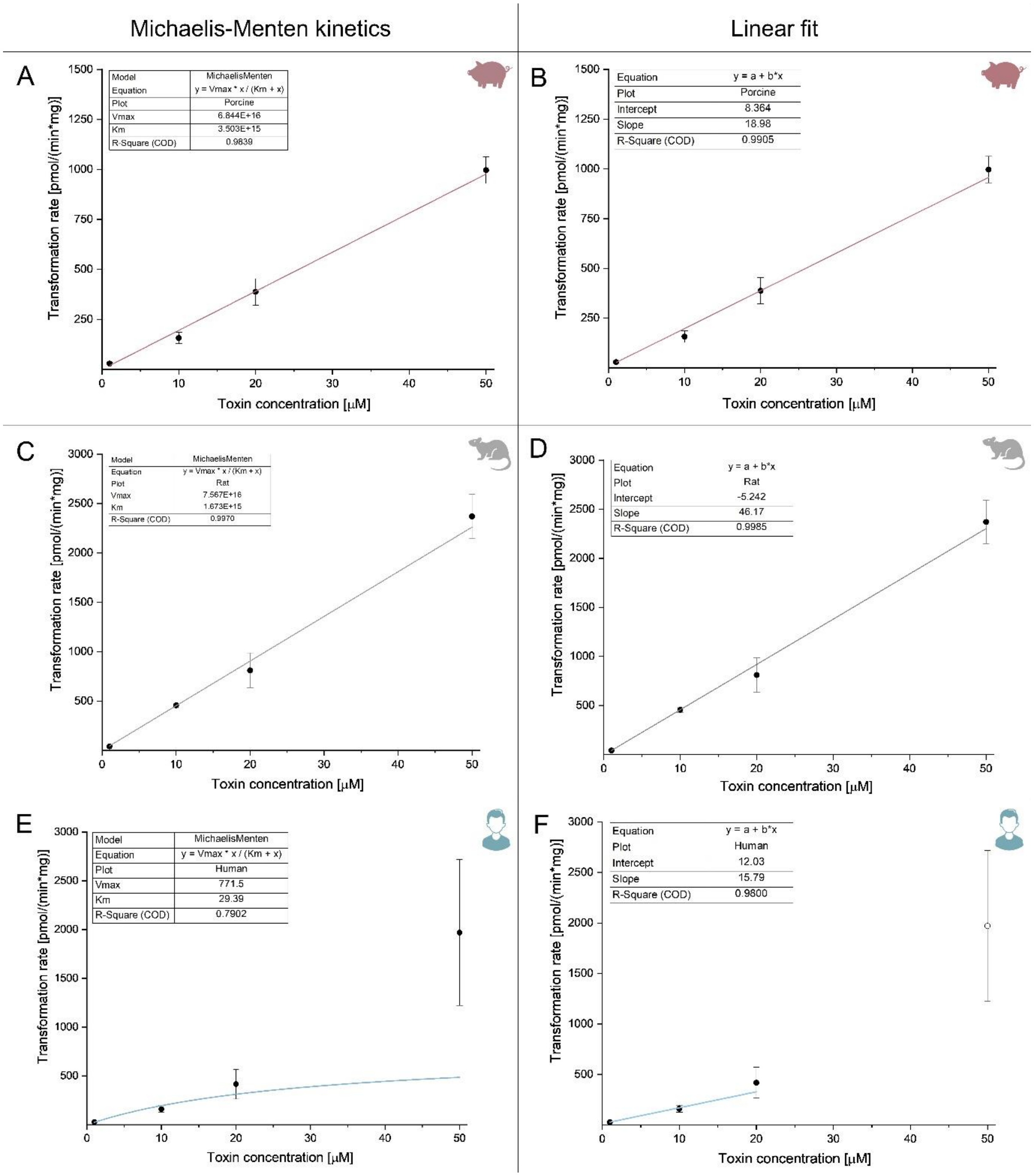

Figure S5. *In vitro* phase I metabolization rates in liver microsomes after 10 minutes of AME exposure. Dots represent means ± SD of at least three independent experiments, with transformation rates normalized to the microsomal protein content plotted against the initial toxin concentration. Data points represented by hollow circles were excluded from the curve-fitting process.

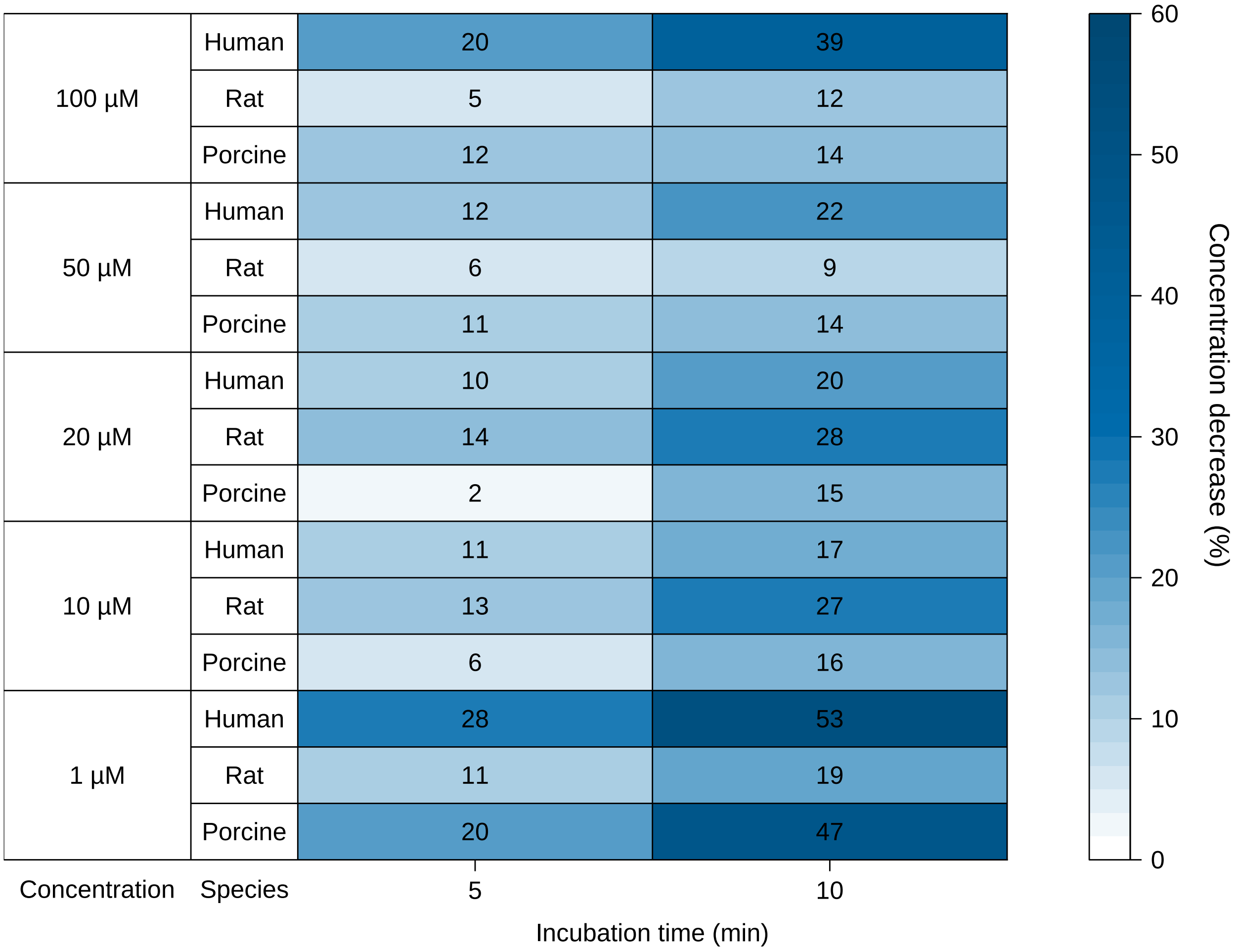

Figure S6: Percentage decrease of AOH in liver microsomes, with an incubation time of 5-10 minutes. Each data was calculated based on the mean AOH concentration of 3-6 independent experiments.

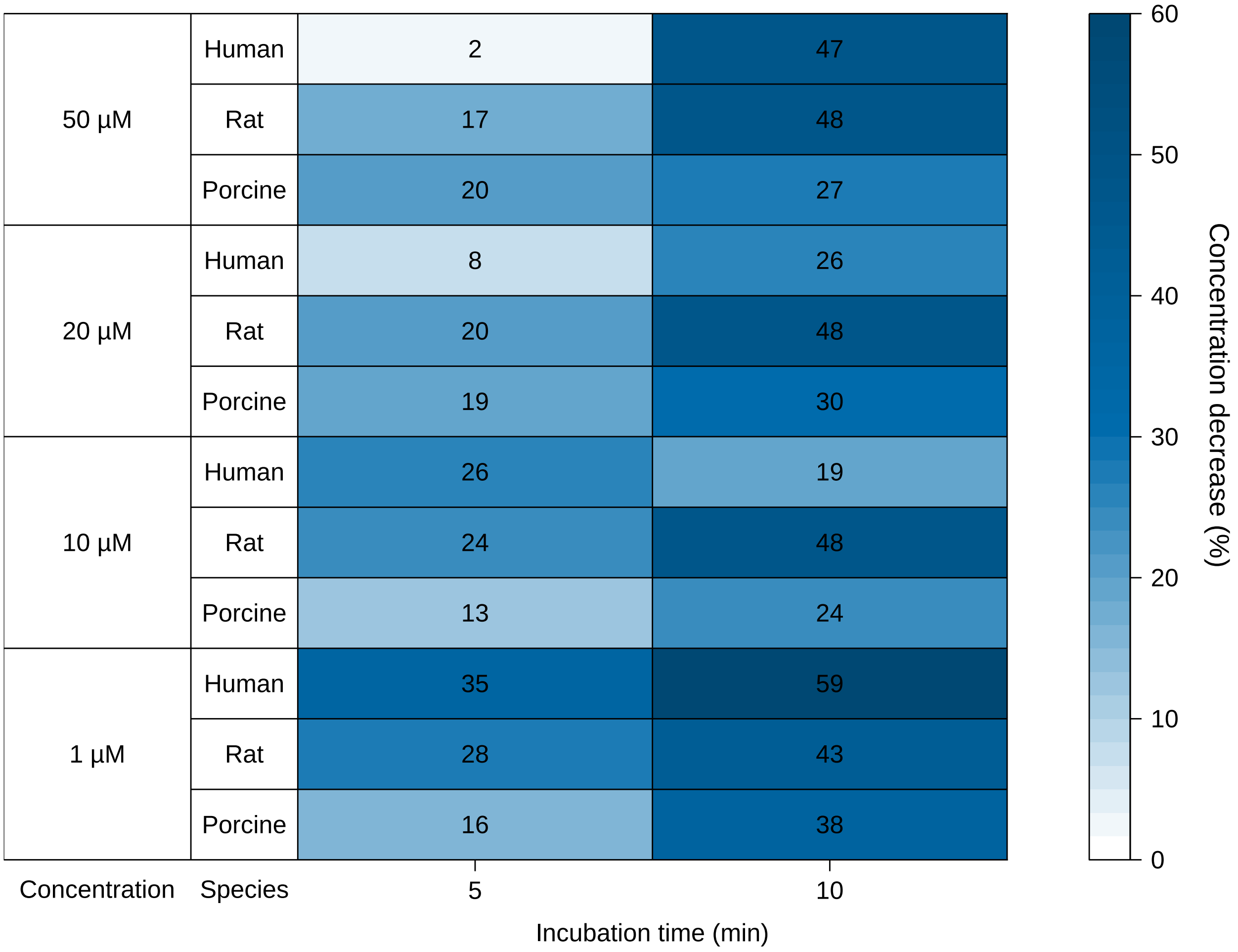

Figure S7. Percentage decrease of AME in liver microsomes, with an incubation time of 5-10 minutes. Each data was calculated based on the mean AME concentration of 3-6 independent experiments.

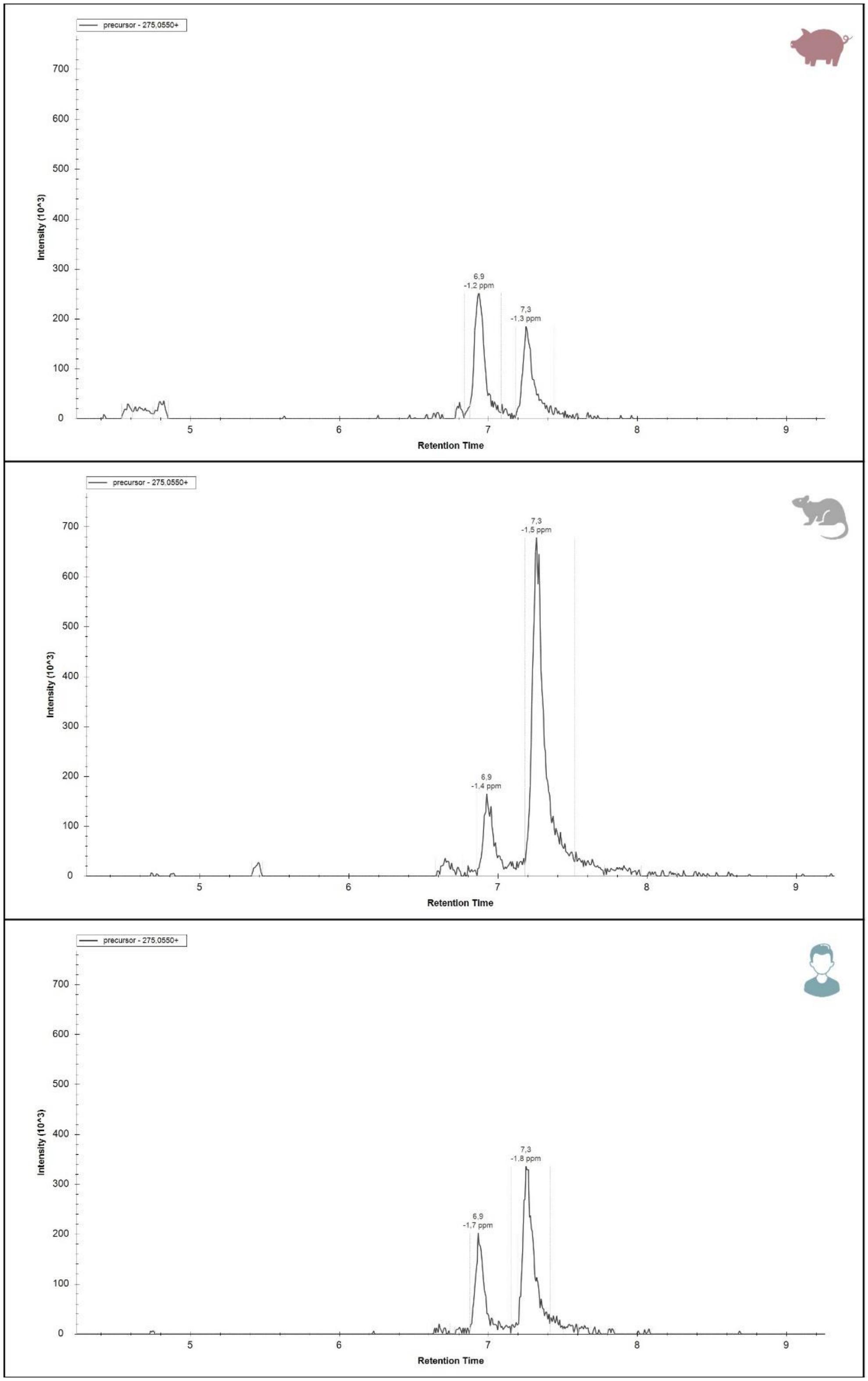

Figure S8: Exemplary chromatograms of monohydroxylated AOH metabolites after a 10-minute incubation of 10 µM AOH with porcine, rat, and human liver microsomes.

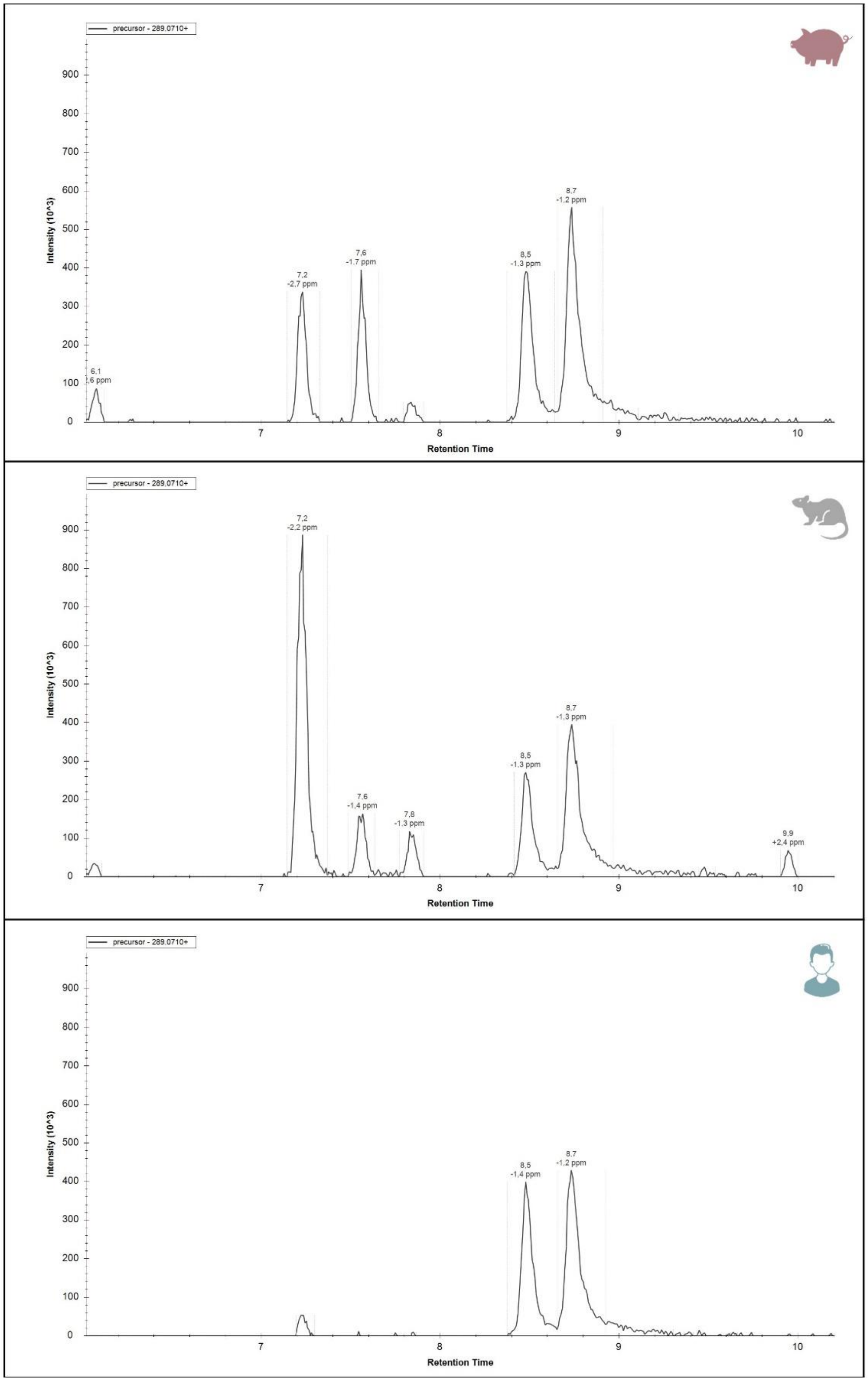

Figure S9: Exemplary chromatograms of monohydroxylated AME metabolites after a 10-minute incubation of 10 µM AME with porcine, rat, and human liver microsomes.
